# Executive Functioning in Daily Life in Parkinson's Disease: Initiative, Planning and Multi-Task Performance

**DOI:** 10.1371/journal.pone.0029254

**Published:** 2011-12-20

**Authors:** Janneke Koerts, Marije Van Beilen, Oliver Tucha, Klaus L. Leenders, Wiebo H. Brouwer

**Affiliations:** 1 Department of Clinical and Developmental Neuropsychology, Faculty of Behavioral and Social Sciences, University of Groningen, Groningen, The Netherlands; 2 Department of Neurology, University Medical Center Groningen, University of Groningen, Groningen, The Netherlands; Federal University of Rio de Janeiro, Brazil

## Abstract

Impairments in executive functioning are frequently observed in Parkinson's disease (PD). However, executive functioning needed in daily life is difficult to measure. Considering this difficulty the Cognitive Effort Test (CET) was recently developed. In this multi-task test the goals are specified but participants are free in their approach. This study applies the CET in PD patients and investigates whether initiative, planning and multi-tasking are associated with aspects of executive functions and psychomotor speed. Thirty-six PD patients with a mild to moderate disease severity and thirty-four healthy participants were included in this study. PD patients planned and demonstrated more sequential task execution, which was associated with a decreased psychomotor speed. Furthermore, patients with a moderate PD planned to execute fewer tasks at the same time than patients with a mild PD. No differences were found between these groups for multi-tasking. In conclusion, PD patients planned and executed the tasks of the CET sequentially rather than in parallel presumably reflecting a compensation strategy for a decreased psychomotor speed. Furthermore, patients with moderate PD appeared to take their impairments into consideration when planning how to engage the tasks of the test. This compensation could not be detected in patients with mild PD.

## Introduction

Motor symptoms are the clinical hallmark of Parkinson's disease (PD), however non-motor symptoms are often present. In particular, cognitive impairments in the domain of executive functioning have frequently been observed [1]–[5], both in late but also in very early stages of the disease [6], [7]. Executive functioning comprises what individuals do, how they do it and whether they do it at all in non-routine situations [7]. It can be defined as deliberate planning and regulation in situations in which previously learned behavioral patterns are not sufficient or not available. It involves inhibition of automated responses, retrieval from declarative memory, planning, monitoring, cognitive flexibility and the maintenance and manipulation of information in working memory [8]. In patients with PD, impairments of working memory, planning, problem-solving, verbal fluency, cognitive flexibility and generating new rules have repeatedly been reported [9]–[14]. Besides these impairments, patients with PD often show a decreased psychomotor speed or bradyphrenia, which may influence executive functioning in daily life.

Executive functioning in daily life is difficult to assess with standard tests focused on executive functions. For example, it has been observed that patients with frontal lobe lesions can suffer from significant impairments in executive functioning in daily life but show an unimpaired performance on standard tests measuring various executive functions [15], [16]. This might result from the fact that available tests used in patient assessment are usually very structured by giving clear rules, setting goals and prompting the start and end of behavior [7], [17]. However, situations in daily life that require executive functioning are usually unstructured, often without a clearly defined goal, solution, start and end. Furthermore, various approaches might be possible to solve a problem in daily life (e.g. planning a journey), while in standardized test procedures there is usually only one correct approach (e.g. the Tower of Hanoi [18] or the ZOO Map of the Behavioral Assessment of the Dysexecutive Syndrome [19]. In addition, these tests are often aimed at measuring a single aspect of executive functioning, such as inhibition or cognitive flexibility, in order to make reliable statements about a function independent from the influences of other functions or their impairment. Executive functioning in daily life, however, requires sustained goal-directed collaboration between the various executive processes. Furthermore, other factors such as mental effort, psychomotor speed and declarative memory may significantly influence executive functioning in daily life. Multi-tasking situations, e.g. talking with someone while walking, are examples of daily life situations involving executive functioning. It has been found that patients with PD show already difficulties in dual-task paradigms [20]–[23]. The general picture emerging from the available literature is that the performance on a primary task deteriorates markedly when a secondary task needs to be performed. It appears that the performance in the primary task deteriorates increasingly with increasing demands of the secondary task [24]. However, the multi-tasking paradigms used in this research provided patients with a lot of structure, and therefore do not resemble daily life.

Considering the above mentioned difficulties, the Cognitive Effort Test (CET; [25]) has recently been developed to give a better reflection of executive functioning in daily life. Compared to tests such as the Tower of Hanoi or the ZOO Map of the Behavioral Assessment of the Dysexecutive Syndrome, the CET not only focuses on *what* participants do or *how* they do it but also on *whether* participants initiate certain behaviors themselves. The CET is a multiple component visual-motor task with proven reliability. It has been validated in patients suffering from schizophrenia with limitations in executive functioning. In contrast to most other tests of executive functioning, the CET is relatively unstructured, giving no clear rules how to engage the tasks of the test. This test provides participants with the opportunity to perform more than one task at the same time if they decide to do so but also allows them to execute the tasks sequentially. Furthermore, participants are left unprepared for (sudden) occurring problems. The CET does not only focus on how and when participants perform a task, but also whether participants perform the task at all. Therefore, the CET provides measures for initiation of behavior, planning and multi-tasking in a relatively unstructured situation.

The aim of the present study is to examine planning and multi-tasking in patients with PD by using the recently developed CET [25]. In addition, it will be investigated to what extent the initiation of behavior, planning and multi-tasking of PD patients are associated with aspects of executive functioning and psychomotor speed as measured with standard test procedures. This study examined and compared patients with mild to moderate PD, since limitations in executive functioning have already been described in early stages of the disease [6].

## Methods

### Ethics statement

This study was approved by the medical ethical committee of the University Medical Center Groningen, The Netherlands. All participants signed an informed consent prior to study inclusion and were treated according to the declaration of Helsinki.

### Participants

Thirty-six PD patients participated in this study. All patients were recruited from the Movement Disorders outpatient clinic of the Department of Neurology of the University Medical Center Groningen and were diagnosed with idiopathic PD according to the criteria of the UK Parkinson's Disease Society Brain Bank. Diagnoses have been made by an experienced neurologist and specialist in the field (K.L.L.). The motor severity of symptoms was assessed with the Unified Parkinson's Disease Rating Scale (UPDRS; [26]) and Hoehn and Yahr scale (H&Y; [27]). A Levodopa Equivalent Daily Dose (LEDD) was calculated for all patients according to the following formula: levodopa dose (100 mg) x 1 (added with 0.2× levodopa dose if using entacapone with each dose) + (slow release levodopa x 0.7) + bromocriptine x 10 + ropinirole x 20 + pergolide x 100 [28]. All patients were assessed in their regular on-state after medication. The patient group consisted of 18 men (50%) and 18 women (50%). In addition, 34 healthy participants were included in this study. The healthy participants were recruited from the Groningen community by means of advertisements in local papers or were related to the patients that participated in this study. This group consisted of 16 men (47%) and 18 women (53%). Level of education was rated for all participants with a Dutch education scale, ranging from 1 (elementary school not finished) to 7 (university degree). Groups did not differ in age (t = -1.07, p = .29), gender (Chi-Square = .06, p = .81) and education level (Mann-Whitney U = 543.00, p = .39). Descriptive and disease characteristics of PD patients and healthy participants are reported in Table 1. Patients with a H&Y stage ≥3 and neurological disorders other than PD have been excluded. Twenty-seven out of the 36 PD patients were assessed with the Mini Mental State Examination (MMSE; [29]) and obtained scores above 24 indicating ‘no dementia’ (M = 27.63, SD = 1.33). For the remaining 9 patients, medical records were checked before inclusion and revealed no indication for the presence of dementia. Exclusion criteria for healthy participants were neurological and psychiatric disorders.

**Table 1:** Descriptive and disease characteristics of PD patients (n = 36) and healthy participants (n = 34).

	PD patients	Healthy participants
	M (SD)	M (SD)
**Age**	60.6 (8.0)	58.8 (6.0)
**Education**	5.6 (0.9)	5.7 (0.9)
**MADRS total**	7.3 (5.5)	3.2 (3.0)
**Disease duration**	4.7 (4.4)	
**UPDRS, part III**	19.2 (6.1)	
**H&Y**	1.9 (0.5)	
**LEDD**	540.0 (520.6)	

MADRS  =  Montgomery-Åsberg Depression Rating Scale; UPDRS  =  Unified Parkinson's Disease Rating Scale; H&Y  =  Hoehn and Yahr scale; LEDD  =  Levodopa Equivalent Daily Dose.

### Stimulus material and procedure

All participants were assessed with the CET. A subgroup of patients (n = 27) and healthy participants (n = 20) also completed a number of standard tests of executive functioning and psychomotor speed. Since the completion of these standard tests was added at a later point in time, not all patients and healthy participants performed these tests. The standard tests applied in this study were selected because there is a considerable body of literature showing that these measures represent standardized, reliable and valid neuropsychological measures [30] and are moreover sensitive to the impairments of patients with neurodegenerative diseases. Furthermore, there are reasonable normative data for the Dutch population available.

#### Cognitive Effort Test (CET)

The CET [25] is a multiple component visual motor test that provides participants with the goal to perform three tasks as accurately and as fast as possible. However, the CET does not offer a structured method that prompts participants to a certain behavior how to approach the different tasks. The test is focused on assessing the initiatives taken by participants, whether and how participants plan to perform the three tasks (sequential or in parallel) and how participants actually perform the three tasks. Participants can voluntarily decide how many tasks they can handle simultaneously in order to complete the CET successfully.

**Figure 1:**
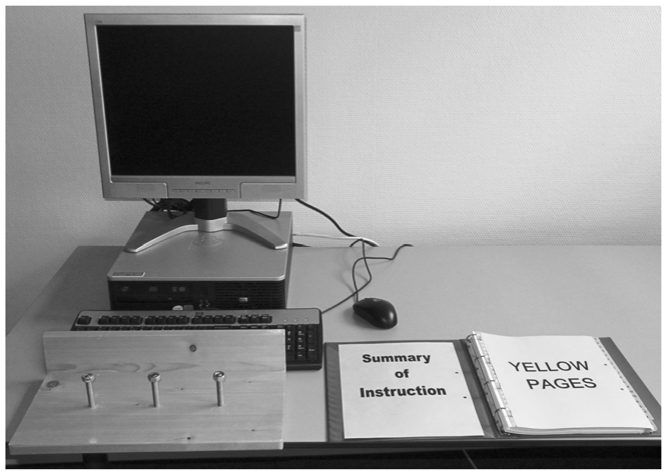
Placement of the tasks of the CET.

The CET consisted of the following three tasks:

1. The Computer task; when started by the participant the computer task runs itself for two minutes during which the participant has to wait before being able to continue. This information is explicitly mentioned in the instruction and is also visible, by means of a timeline presented on the computer screen. At the end of the first minute a textbox appears unexpectedly asking for a password. Since participants were not prepared for this situation and also received no instruction by the instructor, they can show initiative by asking the instructor for the password or by making up a password themselves. However there was only one correct password. At the end of the second minute participants were instructed by a textbox on the computer screen to type the alphabet. After this the computer task was finished, regardless whether any mistakes were made.
2. The Yellow pages task; in this task the participants received a list of five different companies and were asked to find their telephone numbers in a copy of the regional yellow pages. When a telephone number was found, participants were asked to speak the number out loud. Successful solution of this task required focused attention.
3. The Screws task; this task requires participants to thread three nuts up and down three large fixed screws. This is a motor task, which can be performed by either the left hand, the right hand or both. The board to which the screws have been attached had a back wall in a height of eight centimeters (Figure 1).

The objects involved in these tasks are placed in front of the participants in such a way that they allow several initiatives (e.g. moving the screw board, which is blocking the keyboard due to the high back wall, or moving the keyboard; Figure 1).

Instructions: Participants are informed about the tasks and are instructed to complete them as fast and accurately as possible. Furthermore, they received a printed copy of the instructions. Although it is mentioned that they are allowed to do several tasks at the same time, participants are not explicitly instructed to do so. Neither the need of a password nor the necessity to move objects (i.e. the screw board or the keyboard) is mentioned in the instructions. After the instructions are given, the test instructor leaves the room for one minute with an excuse (i.e. searching for a form). Immediately after the return of the instructor, participants are asked how they plan to perform the test.

Three summary scores are computed:

1. Initiative: The participants are rated with regard to the following six initiatives: (1) asking for the password, (2) making up a password themselves, (3) consulting the instruction form, (4) moving the screwboard, (5) moving the keyboard and (6) showing any other initiative during the test (e.g. talking to the test instructor about the test). Each initiative is rated with one point so that a maximum of six points can be obtained with regard to the participants' initiative.
2. Planning: When the test instructor leaves the room participants have the opportunity to form a plan how to solve the test without being prompted to do so. Planning is evaluated in two ways. (1) Making a plan: Participants receive one point when they designed a plan and no points if they failed to do so; (2) Quality of the plan: Participants receive no points when they have planned to do one task at a time, one point when they have planned to do two tasks at the same time and two points when they have planned to do three task simultaneously.
3. Multi-tasking: In order to calculate a multi-tasking score it is important to determine whether participants performed tasks simultaneously when they had the opportunity. Therefore three stages are defined and the proportion of time spent on working on multiple tasks is calculated. (1) In the first stage, it is possible to work on three tasks (i.e. computer task, yellow pages task and screws task). This stage begins when the CET starts and ends when the subject has completed the first task. The proportions of time during which participants work on one or two or even three tasks simultaneously are calculated, adding up to a maximum of 100%. (2) In the second stage, one task is finished and the other two still need to be worked on. The proportion of time working on one or two tasks during this stage is rated, again adding up to a maximum of 100%. (3) In the third stage, two tasks are finished and a maximum of 100% of time is spent on working on this one task.

Subsequently, the total proportion of time during which participants work on one task only is added up over the three stages. Furthermore, the total proportion of time during which participants work on two tasks simultaneously is added up over the two stages during which it is possible (stage 1 and 2). The total proportion of time during which participants work on three tasks simultaneously is the time already calculated during stage 1. This, however, might result in a maximum score of 100% working on three tasks simultaneously, 200% working on two tasks simultaneously and 300% working on one task. If these scores would simply be added, the proportion of time working on one task would have a stronger weight than the proportion of time working on two or three tasks. Similarly, the proportion of time working on two tasks would have a stronger weight than the proportion of time working on three tasks. Consequently, an adjustment is needed to make the scores comparable. Therefore, the total proportion of time working on two tasks is divided by 2 and the total proportion of time working on one task is divided by 3. Finally, these scores are added, which results in a summary score between 100 and 300. A higher score means that participants have performed more time in working on tasks simultaneously. An example of this calculation is given in Table 2.

**Table 2:** Two examples of calculating multi-tasking score.

	Example 1		Example 2	
Total time CET	420 s		480 s	
*Stage 1*				
Total time	180 s		55 s	
Time on 3 tasks simultaneously	20 s	11%	0	0%
Time on 2 tasks simultaneously	100 s	55%	0	0%
Time on 1 task	60 s	33%	55	100%
*Stage 2*				
Total time	215 s		225 s	
Time on 2 tasks simultaneously	55 s	26%	0 s	0%
Time on 1 task	160 s	74%	225 s	100%
*Stage 3*				
Total time	25 s		200 s	
Time on 1 task	25 s	100%	200 s	100%
Proportion of time spent on 3 tasks simultaneously	11%	11.00	0%	0.00
Proportion of time spent on 2 tasks simultaneously	(55%+26%)/1.5	53.72	(0%+0%)/1.5	0.00
Proportion of time spent on 1 task	(33%+74%+100%)/3	69.14	(100%+100%+100%)/3	100.00
Total Multi-tasking		133.86		100.00

#### Standard tests of executive functions

The *Stroop Color Word Test* [31] is used to assess the ability of a person to maintain a goal in mind and to suppress a habitual response in favor of a less familiar one (i.e. inhibition). This test contains three cards, the Word card, the Color card and the Color-Word card. The target measure for inhibition is the Color-Word card. This task requires participants to suppress the automatic tendency to read, while naming the color of words that are themselves color names. The performance was corrected for psychomotor speed, by dividing the time needed for the Stroop Color-Word card by the time needed for the Stroop Color card.

Cognitive flexibility was assessed with the *Trail Making Test* [32] which consists of two parts. Part A requires participants to draw a line, as fast as possible, between numbers in ascending order. In part B, numbers and letters are used and participants need to switch attention between both concepts: they have to draw a line between both types of stimuli in ascending order, alternating between numbers and letters and also as fast as possible. The target measure for cognitive flexibility was the performance on part B.

The *Odd Man Out* [33] was also used to measure cognitive flexibility. This test requires participants to indicate which shape, out of a set of four shapes, is different. Three selection rules are possible and two sets of twelve cards are used. For each set of cards participants have to specify a different rule and both sets are alternated four times. The total number of incorrect responses is calculated.

The *Digit Span* of the Wechsler Memory Scale–Revised [34] was used to assess verbal short term and verbal working memory. A series of digits are read to the participants who are required to repeat the digits either in the given order or in the reverse order.

*Semantic and phonemic verbal fluency tests* were used to evaluate the ability to spontaneous produce words under restricted search conditions. The semantic verbal fluency test requires participants to produce as many animals or professions as possible, each within a time interval of one minute. Participants are not allowed to name the same word twice. The total number of correctly produced animals and professions is registered. During the phonemic verbal fluency test, participants are asked to produce as many words as possible within one minute starting with the letters D, A or T [35]. This verbal fluency test is equivalent to the FAS-test as devised by Benton & deHamser [36]. Participants are not allowed to name the same word twice or to produce names of persons or towns. The total number of correctly produced words starting with D, A and T were calculated and combined into one phonemic verbal fluency score.

The time needed for completion of Part A of the Trail making test [32] and of the Stroop Word Card [31] were used as a measure of psychomotor speed.

### Statistical analyses

Tests of normality of data indicated that not all variables were normally distributed. Therefore, non-parametric tests were used for analysis. The Mann-Whitney U test was used to compare the performance of PD patients and healthy participants on the CET, psychomotor speed tests and the standard tests for executive functioning. To determine the associations between the performance on the CET and these tests for executive functioning and psychomotor speed, Spearman rank correlations were calculated within groups. Besides comparing PD patients to healthy participants, the performance of PD patients in H&Y stage 1–1.5 on the CET was compared to the performance of PD patients in H&Y stage 2–2.5, using the Mann-Withney U test. These groups were also compared with regard to their performance on the remaining tests. Based on previous literature one-tailed p-values have been chosen for analyses [1], [6], [37], [38]. Cohen's d was calculated for all comparisons between groups [39]. Furthermore, where possible patients' test performances were compared to published normative data. This method is used in clinical practice and increases external validity. Standard scores and if possible percentiles were derived for each patient, using published normative data of healthy individuals (Stroop [40], Trail Making Test [40], Odd Man Out [41], Digit Span [42], Semantic Fluency [43] and Phonemic Fluency [35]). All normative data sets included a correction for age and when relevant a correction for gender and level of education. According to Lezak [30] cognitive impairment on a test was defined as a performance equivalent to the performance of the lowest 10% of the normative samples as provided by the test authors.

## Results

PD patients showed significantly lower scores on the multi-tasking and the quality of the plan measures of the CET than healthy participants. No differences were found between groups with regard to the initiative and the making a plan measures (Table 3). PD patients also showed significantly lower scores on psychomotor speed (Trail Making A Test) and standard tests assessing cognitive flexibility (Trail Making B Test and Odd Man Out test). A trend toward a significant difference was found for working memory (Digit Span backwards; Table 4). A more clinical approach which compares the performance of PD patients with published normative data revealed that only a small number of PD patients showed impairments on standard tests of executive functioning and psychomotor speed (Table 4).

**Table 3:** Comparison of Cognitive Effort Test scores of PD patients (n = 36) and healthy participants (n = 34; one-tailed).

	PD patients	Healthy participants			
	M (SD)	M (SD)	Z	p	d
**Initiative**	2.6 (1.1)	3.0 (1.2)	−1.0	.164	.35
**Making a plan**	.9 (.3)	.8 (.4)	−1.2	.124	.29
**Quality of plan**	.7 (.5)	1.1 (.7)	−2.9	.002**	.67
**Multi-tasking**	114.1 (10.9)	125.0 (18.9)	−2.4	.008**	.73

**p<.01.

**Table 4:** Performance of PD patients (n = 27) and healthy participants (n = 20) on standard tests of executive functions and psychomotor speed (one-tailed).

	PD patients		Healthy Participants			
	M (SD)	n (%)^#^	M(SD)	Z	p	d
**Executive functions**						
Stroop interference index	1.6 (.3)	2 (7)	1.5 (.2)	−.9	.172	.40
TMT B	97.0 (35.4)	3 (11)	70.2 (24.4)	−3.0	.002**	.90
OMO no errors	3.1 (4.1)	0 (0)	1.2 (2.0)	−2.6	.005**	.63
Fluency animals	24.3 (4.4)	0 (0)	23.4 (4.6)	−.3	.393	−.20
Fluency professions	17.4 (3.4)	1 (4)	19.5 (5.5)	−1.8	.039*	.48
Fluency letters	39.8 (12.5)	3 (12)	44.8 (10.8)	−1.2	.117	.43
WMS digit span backwards	5.9 (1.7)	1 (4)	7.2 (2.5)	−1.6	.053	.62
**Psychomotor speed**						
Stroop word card	48.2 (9.7)	7 (26)	49.8 (13.0)	−.4	.361	−.14
TMT A	45.3 (15.6)	5 (19)	37.6 (13.8)	−1.8	.040*	.55

*p<.05.

**p<.01.

# number and percentage of patients impaired; TMT  =  Trail making test; OMO  =  Odd man out test; WMS  =  Wechsler Memory Scale.

Correlational analyses showed that within the group of PD patients quality of the plan and multi-tasking were significantly associated with cognitive flexibility (Trail Making B Test), one of the fluency measures (Fluency professions) and psychomotor speed (Trail Making A Test and Stroop Word card; Table 5). Within the group of healthy participants multi-tasking was significantly associated with cognitive flexibility (Trail Making B Test; Table 5). Since performance on both the verbal fluency test (Fluency professions) and Trail Making B test are influenced by psychomotor speed, the correlations between planning, multi-tasking and these variables may have been mediated by psychomotor speed. Therefore, partial correlations were calculated between planning and multi-tasking and Trail Making B test and Fluency professions, while controlling for psychomotor speed (i.e. a combined score of Trail Making A test and Stroop Word card). In PD patients, the correlations between Trail Making B test and both planning (r = −.09, p = .484) and multi-tasking (r = −.12, p = .294) were no longer significant. Furthermore, the association between planning and fluency professions did not reach significance anymore (r = .26, p = .120). However, following correction a trend could still be found with regard to the correlation between multi-tasking and verbal fluency (Fluency professions) in patients with PD (r = .32, p = .067). Correlation analysis between the performances of healthy participants in the Trail Making B Test and the multi-tasking measure also resulted in a trend toward a significant association (r = −.39, p = .077).

**Table 5:** Spearman correlations coefficients between CET quality of the plan and multi-tasking and standard tests of executive functions and psychomotor speed in healthy participants (n = 34) and PD patients (n = 36).

	PD patients		Healthy participants	
	Quality of the plan	Multi-tasking	Quality of the plan	Multi-tasking
**Executive functions**				
Stroop interference index	−.02	.12	−.09	−.29
TMT B	−.61**	−.59**	−.33	−.45*
OMO no errors	−.18	−.10	−.02	.16
Fluency animals	.37	.16	.06	.08
Fluency professions	.53**	.52**	−.10	−.24
Fluency letters	.38	.07	−.08	−.12
WMS digit span backward	.25	.09	.24	.26
**Psychomotor speed**				
Stroop word card	−.62**	−.51**	−.12	.10
TMT A	−.60**	−.45**	−.21	−.11

*p<.05.

**p<.01; TMT  =  Trail making test; OMO  =  Odd man out test; WMS  =  Wechsler Memory Scale.

Besides comparing PD patients to healthy participants, the performance of PD patients in H&Y stage 1–1.5 on the CET was compared to the performance of PD patients in H&Y stage 2–2.5. PD patients in H&Y 2–2.5 showed significantly lower scores on the quality of the plan variable than PD patients in H&Y stage 1–1.5. No differences were found between these groups with regard to the initiative and multi-tasking scale and the making a plan variable (Table 6). PD patients in H&Y stage 2–2.5 also showed significantly higher scores than PD patients in H&Y stage 1–1.5 on standard tests measuring inhibition (Stroop interference index) and cognitive flexibility (Trail Making B Test and Odd Man Out test; Table 7). On the basis of normative data, more patients with moderate PD were classified as being impaired in standard measures of executive functioning and psychomotor speed than patients with a mild PD (Table 7).

**Table 6:** Cognitive Effort Test scores of PD patients in H&Y stage 1 and 1.5 (n = 12) compared to PD patients in H&Y stage 2 and 2.5 (n = 24; one tailed).

	PD patients in H&Y 1–1.5	PD patients in H&Y 2–2.5			
	M (SD)	M (SD)	Z	p	d
**Initiative**	2.9 (1.2)	2.5 (1.1)	−1.0	.153	.35
**Making a plan**	.9 (.3)	.9 (.3)	.0	.500	.00
**Quality of plan**	.9 (.5)	.6 (.5)	−1.7	.041*	.60
**Multi-tasking**	117.8 (9.8)	112.2 (11.3)	−1.2	.113	.53

*p<.05; H&Y  =  Hoehn and Yahr stage.

**Table 7:** Performance of PD patients in H&Y stage 1–1.5 (n = 8) compared to PD patients in H&Y 2–2.5 (n = 19) on standard tests of executive functions, memory and psychomotor speed (one-tailed).

	PD patients H&Y 1–1.5		PD patients H&Y 2–2.5				
	M (SD)	n^#^	M(SD)	n^#^	Z	p	d
**Executive functions**							
Stroop interference index	1.4 (.2)	0	1.7 (.3)	2	−1.8	.040*	1.20
TMT B	77.0 (11.5)	0	105.4 (38.8)	3	−2.2	.015*	1.14
OMO no errors	1.4 (2.0)	0	3.8 (4.5)	0	−2.0	.023*	.72
Fluency animals	23.1 (4.0)	0	24.7 (4.5)	0	−1.0	.169	.38
Fluency professions	16.8 (3.3)	0	18.8 (3.4)	1	−1.3	.100	.59
Fluency letters	41.3 (11.8)	1	39.3 (13.1)	2	−.2	.405	.16
WMS digit span backwards	5.9 (1.9)	1	6.0 (1.7)	0	−.3	.393	.06
**Psychomotor speed**							
Stroop word card	45.9 (4.0)	1	49.1 (11.3)	6	−.3	.395	.14
TMT A	38.6 (2.9)	0	48.1 (17.9)	5	−1.3	.099	.91

*p<.05.

# number of patients impaired; H&Y  =  Hoehn and Yahr stage; TMT  =  Trail making test; OMO  =  Odd man out test; WMS  =  Wechsler Memory Scale.

## Discussion

PD patients showed significantly lower scores than healthy participants on both the quality of the plan measure and the multi-tasking measure. This finding indicates that PD patients plan and demonstrate sequential task execution instead of parallel task execution. Impairments in planning [44], [45] and dual-tasking [20]–[23] in PD might explain these results. However, both measures (quality of the plan, multi-tasking) were significantly associated with a decreased psychomotor speed. None of the executive functions examined were significantly associated with the quality of the plan, while multi-tasking was only associated with semantic verbal fluency. These results suggest that PD patients with a more pronounced psychomotor slowing planned and also demonstrated sequential task execution more often. Since a decreased psychomotor speed or bradyphrenia is one of the main characteristics of PD, it appears that planning and demonstrating sequential task execution may be a wise strategy to compensate for psychomotor slowing. This is in line with a previous study focused on planning in PD which also used a test reflecting daily life performance (a computerized cooking task [46]). It was found that PD patients and healthy participants approached the test with different strategies. While healthy participants performed two tasks at the same time, PD patients improved their performance by focusing on one task and spending less time on the other task. Patients with PD appear thus to take their impairment of being slower into consideration and cope with this by planning and demonstrating more sequential task execution. The finding that semantic fluency predicted multi-tasking is not surprising considering that fluency measures reflect divergent thinking which has often been seen as a measure of creativity [47]. Divergent thinking appears to be crucial in multi-task situations specifically for the disengagement from current solutions and the development of alternative solutions [48]. Consequently, PD patients who show difficulties with divergent thinking are also very likely to suffer from problems in multi-tasking situations of everyday life.

The performance on the CET differs, however, between PD patients at different stages of the disease. Patients with a mild PD planned the execution of the same number of tasks at the same time as healthy participants (i.e. there was no difference between these groups with regard to the quality of the plan measure). PD patients with a moderate disease severity, however, showed a significantly lower score on the quality of the plan measure as compared to patients with a mild PD. This implies that only patients with a moderate PD take their impairments into consideration when planning the execution of the different tasks of the CET. Several longitudinal studies reported that PD patients showed a faster rate of cognitive decline than matched healthy participants [37], [49], particularly in the domains of attention and psychomotor speed [38]. However, if patients indeed compensate for slow psychomotor speed, one would expect subjective insight in their cognitive impairments. Little is known about the subjective experience of PD patients with regard to their cognitive impairments and their adaptations for these restrictions in daily life. The subjective experience of cognitive impairments of PD patients has, however, not been investigated in detail, even though it is clear that cognitive impairments have a negative influence on quality of life of patients with PD [50]. Knowledge about patients' subjective experience or awareness of cognitive impairments is crucial in clinical neurological practice. If PD patients are not aware of their cognitive impairments they do not report them to the attending neurologist. Consequently, cognitive impairments may not always be treated in PD patients even though this treatment may be helpful in improving their daily life functioning.

The present results also indicate that PD patients and healthy participants did not differ on the initiative measure of the CET. Patients were also not hindered by their motor symptoms in showing initiatives. They moved the screw board and the keyboard as often as healthy participants in order to execute the CET. Previous studies reported that PD patients showed difficulties initiating behavior when they are not provided with an external cue [51], [52]. However, studies that focused on externally cued or internally motivated initiatives often used reaction times as an outcome measure. Unfortunately, it is usually not directly investigated whether participants show initiatives or not. The latter is, however, assessed by the Initiative measure of the CET and may therefore explain why in this study no differences were found between PD patients and healthy participants. However, this finding has to be viewed with caution since a non-significant result does not mean that there is no difference.

Finally, in healthy participants only a trend toward a significant association was found between the Trail Making B Test and multi-tasking. All other standard test measures were not associated with the CET. Based on these findings the question can arise whether the CET is a helpful measure in the assessment of executive functioning. In this context it appears to be important to consider that standard neuropsychological tests as applied in the present study were designed to assess brain pathologies. Consequently, these measures may not reliably depict variability of functioning of healthy participants. Furthermore, a lack of correlations indicates that the CET measures aspects of executive functioning which are not assessed by the more standard neuropsychological tests. As already stated in the introduction, the CET is different in structure from other tests of executive functions (e.g. measuring more than one aspect of functioning, no clear rules, opportunity for multi-tasking and it focuses not only how and when but also whether a task is performed). It is thus not surprising that there is a lack of correlations between the CET measures and measures of executive functions and psychomotor speed in healthy participants.

A limitation of this study was the relatively small group of patients with a mild disease severity. The effect sizes of the different comparisons indicate that more significant differences would have been found between the disease severity groups if a larger patient sample had been included. Furthermore, multiple comparisons were performed within this study. This strategy increases the likelihood of type I error. The significant differences of the present study are however consistent with effect sizes and all differences and correlations found to be significant were of medium to large size. Another limitation is that not all of the patients were screened for dementia by using a standardized test. Furthermore, healthy participants were not screened for mild cognitive impairment before inclusion. Therefore, it cannot be excluded that some of the healthy participants might have suffered from mild cognitive impairments. However, in case that some healthy participants might have suffered from cognitive impairment, this would make the present findings concerning an impaired multi-tasking in PD even more conservative. A final limitation of this study was that not all participants were assessed with the full battery of tests.

In summary, patients with PD take their psychomotor slowing into consideration when planning and execution tasks. They cope with their impairment by planning and demonstrating more sequential task execution. This adaptation is especially present in patients with a moderate PD and was not detected in patients with a mild disease severity. Future studies on cognition in PD should not only focus on the quantification of cognitive impairments. For treatment purposes it is also important to determine whether and to what extent PD patients are aware of their cognitive impairments and whether they can adapt their behavior accordingly.
